# Family management characteristics in parents of children with retinoblastoma: a latent profile analysis

**DOI:** 10.3389/fped.2025.1633076

**Published:** 2026-01-20

**Authors:** Changjuan Zeng, Lingling Zhou, Peixia Wu, Ting Zhao, Guanghao He, Hui Wang, Lin Wang, Renbing Jia, Lili Hou

**Affiliations:** 1 Shanghai Jiao Tong University School of Nursing, Shanghai, China; 2 Ninth People’s Hospital, Affiliated to Shanghai Jiao Tong University School of Medicine, Shanghai, China; 3 Shanghai Key Laboratory of Orbital Diseases and Ocular Oncology, Shanghai, China; 4 Xinhua Hospital, Affiliated to Shanghai Jiao Tong University School of Medicine, Shanghai, China; 5 Eye & ENT Hospital of Fudan University, Shanghai, China; 6 College of Mechanical Engineering, Zhejiang University of Technology, Hangzhou, China

**Keywords:** children, family management, family nursing, latent profile analysis, neoplasms, retinoblastoma

## Abstract

**Background and aim:**

The effective management of a child with cancer by their family depends on their coping strategies, the child's treatment outcomes, and their quality of life. For families of children with retinoblastoma, this study aims to use latent profile analysis to categorize family management patterns, understand their traits, explore influencing factors, and provide a theoretical basis for targeted interventions.

**Methods:**

From February to April 2024, a cross-sectional study was conducted with the parents of 608 children with retinoblastoma. These parents completed a survey assessing family management, comprehensive needs, coping tendencies, family functioning, social support and levels of depression, anxiety and stress.

**Results:**

Three family management categories were defined as: high-level (*n* = 93), moderate-level (*n* = 268), and low-level functioning (*n* = 247). Multiple logistic regression analysis showed that better family functioning (odds ratio [OR] = 0.821, *P* = 0.004), unilateral diseased eyes (OR = 0.286, *P* = 0.001) and high social support (OR = 0.972, *P* = 0.023) increased the likelihood of high-level functioning group. Factors linked to the low-level family management group included severe depression (OR = 1.320, *P* = 0.005), severe stress (OR = 1.210, *P* = 0.033), high comprehensive needs (OR = 1.025, *P* = 0.001), junior high school and below education (OR = 4.021, *P* = 0.005).

**Conclusions:**

The family management characteristics of parents of children with retinoblastoma exhibit group heterogeneity, and key factors affecting this heterogeneity have been identified. These include family functioning, comprehensive needs, educational background, depression, stress, and social support. Healthcare professionals can use this information to develop targeted intervention strategies and improve family management.

## 1 Introduction

Retinoblastoma (RB) is the most common primary intraocular malignancy in children under 5 years old and one of the six childhood cancers prioritized by the World Health Organization Global Initiative on Childhood Cancer (1). The one-year postoperative survival rate for children with RB in Africa is 78.2%, and the three-year survival rate reaches 66.2% (2). In industrialized countries, survival rates have risen to >95%–98% (3). Despite these improvements, RB treatment is often prolonged and requires continuous follow-up, placing a significant burden on families. RB survivors report poorer health-related quality of life compared to their siblings, with negative impacts across almost all health domains (4). As cancer care shifts towards outpatient treatment and home care, family caregivers have become central to the support system for children with cancer (5). This shift demands more emotional investment, time, and energy from families to fully meet the child's needs during recovery (6). Therefore, it is crucial to enhance societal understanding and support for families of children with RB, building a comprehensive support system for both children and their families.

Family function is closely linked to caregivers' quality of life (7). Assessing the health and well-being of both patients and family members reveals that cancer significantly impacts both groups, often affecting family members more than the patients themselves (8). Parents of children with cancer report a substantial decline in their quality of life, strongly associated with various dimensions of family function (9). Given the serious challenges in pediatric oncology, effective family management is crucial for enhancing the family's ability to combat the disease (10). Efficient home management by caregivers positively correlates with the quality of life of chronically ill children (11). The developmental outcomes of childhood illnesses are closely related to family management, with positive management patterns significantly associated with both family and child functional performance (12). Therefore, improving home management efficacy for children with tumors is essential to home care.

The Family Management Style Framework(FMSF) offers a systematic approach for families to organize, integrate, and accomplish management tasks associated with chronic disease (13). This framework applies not only to families of children with chronic illnesses but also to broader health domains, including families with adult members facing health challenges (14). Notably, the FMSF has shown good adaptability and effectiveness in Chinese families, emphasizing the importance of improving family management through intervention (15). This improvement benefits the quality of life of the child while providing essential support for the entire family. Parents play an integral role in managing the physical health, emotional, and psychosocial well-being of their children with tumors, serving as their primary source of social and emotional support as well as aiding them in effectively managing their disease (16). Accurately identifying different categories of home management among parents of children with RB is crucial for implementing targeted and individualized intervention strategies. Latent profile analysis (LPA) is an individual-centered statistical method that classifies cases based on probability estimation and comparison. It can categorize samples into distinct groups based on various characteristics and explain associations between external continuous variables and latent category variables (17). This study aims to explore potential categories, characteristics, and influencing factors of LPA-based family management for parents of children with RB, providing a valuable theoretical basis for developing rational interventions. By facilitating the early identification of family management characteristics, this research will aid healthcare providers in understanding the underlying influencing factors.

## 2 Design and methods

### 2.1 Participants

A convenience sampling method was used to survey parents of children with RB who had been treated as inpatients or outpatients in ophthalmology departments at the Ninth People's Hospital affiliated with Shanghai Jiao Tong University School of Medicine, Eye & ENT Hospital of Fudan University, and the Xinhua Hospital affiliated with Shanghai Jiao Tong University School of Medicine, from February to April 2024. The study was approved by the hospital's Ethics Committee [grant number: SH9H-2024-T18-1]. All participating parents provided informed consent and voluntarily took part in the investigation.

**Inclusion criteria:**

Children: clinically diagnosed with RB; no severe organic pathology.

Parents: father or mother of the child with RB; primary caregiver for the child's schooling, daily living, or medical care; familiar with the child's basic condition; able to read and communicate normally.

**Exclusion criteria:**

Children: comorbidity with other severe diseases.

Parents: unable to complete the investigative tools and questionnaires.

### 2.2 Sample size

According to sample size calculation principles, the required sample size should be 5–10 times the number of variables in an analytical study (18). With 52 variables and accounting for a 20% questionnaire invalidity rate, the required sample size ranged from 312 to 624. This study collected 619 questionnaires, of which 608 were valid, yielding an effective response rate of 98.22%.

### 2.3 Investigative tools

#### 2.3.1 General information questionnaire

Based on a literature review and group discussions, a general information questionnaire was designed with two parts: (1) Child's information: age, duration of disease, gender, laterality of diseased eyes (unilateral or bilateral), and family history of disease. (2) Parents' information: age, duration of care, relationship with child, residence, education level, number of children, type of family (nuclear family, stem family, other types), and caregiving type (independent care, assisted care).

#### 2.3.2 Family management measure (FaMM)

FaMM was adapted to Chinese by Ying Zhang et al. (19) to assess how families of chronically ill children manage the illness, the caregiver's management level, and the impact on daily family life. It consists of 53 items across six subscales: child's daily life, condition management ability, parent mutuality, condition management effort, family life difficulty, and view of condition impact. A 5-point Likert scale is used, with responses ranging from 1 to 5, representing “strongly disagree,” “disagree,” “unsure,” “agree,” and “strongly agree,” respectively. Among them, the child's daily life, condition management ability, and parent mutuality are facilitating factors for family management, where higher scores indicate better management. Condition management effort, family life difficulty, and view of condition impact are hindering factors, where higher scores indicate more difficulty in managing the child's illness.

#### 2.3.3 Comprehensive needs assessment tool in cancer for caregivers

Developed by Korean scholars in 2011 and adapted into Chinese by Xinshang Zhao (20), this scale assesses the comprehensive needs of caregivers of patients with cancer. There are 41 items, each of which is rated on a scale of 0–3. “0” represents “no need”, “1” represents “low need”, “2” represents “medium need” and “3” represents “high need”. The total score ranges from 0 to 123. Higher scores indicate a greater need for support from caregivers.

#### 2.3.4 Family APGAR Index questionnaire

Developed by American scholars Smilkstein, G. et al. (21) and revised by Lv Fan et al. (22), this scale consists of five factors: adaptability, cooperation, adulthood, emotionality, and intimacy. A 3-point Likert scale is used, with scores ranging from 0 to 2 for each factor based on responses from “almost rarely” to “often,” resulting in a total score of 0–10. Higher scores indicate better family functioning.

#### 2.3.5 Depression-anxiety-stress scale 21 items (DASS-21)

The DASS-21 measures the severity of negative emotions, including depression, anxiety, and stress, and (or) the corresponding physiological responses during the past week (23). The scale comprises three subscales (depression, anxiety, and stress), each consisting of 7 items rated on a 4-point Likert scale (0–3) with scores indicating frequency from “does not meet” to “always meets.” The total score for each subscale ranges from 0 to 21. Higher scores indicate a greater presence of negative emotions relating to depression, anxiety and stress, respectively.

#### 2.3.6 Simplified coping style questionnaire (SCSQ)

The SCSQ is a self-assessment scale simplified and modified from the foreign coping styles scale by Xie Yaning (24). It measures various attitudes and strategies individuals commonly adopt in their daily lives. The scale consists of 20 items: the first 12 items assess positive coping characteristics, while the last 8 items evaluate negative coping characteristics. Responses are scored as follows: “don't use” (0), “occasionally use” (1), “sometimes use” (2), and “often use” (3), respectively.

#### 2.3.7 Perceived social support scale (PSSS)

The PSSS, compiled by Zimet et al., assesses perceived support from family, friends, and social networks. It includes 12 items across three dimensions: family support (4 items), friend support (4 items), and other support (4 items). Each item is rated on a 7-point Likert scale, with total scores ranging from 12 to 84. Higher scores indicate higher levels of perceived social support (25).

### 2.4 Data collection and quality control methods

The project leader posted the recruitment announcement in a WeChat group for parents of children with RB, explaining the purpose, content and significance of the study. The project leader strictly adhered to the established inclusion and exclusion criteria when screening eligible respondents. After obtaining consent, the online questionnaire was administered via the Questionnaire Star platform (https://www.wjx.cn/). The survey was conducted anonymously, with one submission permitted per IP address only, to prevent duplicate responses. After data collection, the project leader exported the data and applied a two-person review process to eliminate errors and ensure reliable results.

### 2.5 Statistical analysis

Data entry was conducted using Excel 2016, while Mplus 8.3 software analyzed the potential profile model of family management for parents of children with RB. The six subscales from FaMM served as exogenous variables. Models with subjects from the entire single group, vs. models with the best fit for subjects divided into two groups, three groups, or four groups, were created. The four model profiles were assessed by comparing fit indices to identify the optimal model: (1) information indicators such as the Akaike information criterion (AIC), Bayesian information criterion (BIC), and adjusted Bayesian information criterion (aBIC), were used to determine model fit (good or bad), with smaller values indicating better fit; (2) classification indicators included entropy, ranging from 0 to 1, where ≥0.8 indicate 90% classification accuracy, with values closer to 1 reflecting greater accuracy; (3) Likelihood ratio test metrics, including the Lo-Mendell-Rubin (LMR), corrected likelihood ratio test and Bootstrap-based likelihood ratio test (BLRT), were considered significant at (*P* < 0.05), indicating that the k-category model significantly outperformed the k-1 category model. The optimal model was identified by the smallest AIC, BIC, and aBIC, an entropy value >0.7, and statistically significant LMR and BLRT results (*P* < 0.05).

SPSS 25.0 was used for the statistical analysis. Measurement data were expressed as the median (quartiles), and count data as frequency and percentage. Statistically significant variables were identified through Fisher's Exact Test, Pearson Chi-Square Test, or Kruskal–Wallis H Test. Multivariate logistic regression was used to analyze the influencing factors of different categories of family management characteristics among parents of children with RB, with *P* < 0.05 considered statistically significant.

## 3 Results

### 3.1 Basic characteristics of children with RB and their parents

In this study, there were a total of 608 parents of children with retinoblastoma interviewed: The children's average age was 52.15 ± 30.16 months, with a disease duration of 30.18 ± 25.94 months. The gender ratio of boys to girls was nearly 1:1, and the ratio of monocular to binocular cases was about 2:1. Only 2.47% had a family history of RB. The average age of parents was 34.37 ± 5.20 years, with a mean caregiving duration of 7.82 ± 3.87 h. The number of mothers participating in the survey was about twice that of fathers. The urban-to-rural residence ratio was close to 1:1, and individuals with a college education or higher made up about half of the participants. Single-child and two-child families predominated, while families with three or more children comprised 13.16%. Nuclear families accounted for 50.16%, and shared care by others was the primary caregiving type (70.07%). The ratio of positive to negative coping strategies was also near 1:1 (see Table 2).

### 3.2 Naming of family management characteristic categories of parents of children with RB

We explored potential profile models of categories 1 to 4 using the scores from six subscales of FaMM for parents of 608 children with RB as exogenous variables. The fitting results for each model are shown in Table 1. For the 3-category model, the entropy value was 0.844, and the *p*-values for the LMR and the BLRT were statistically significant (*P* < 0.05). In contrast, while the 4-category model had the smallest values for the AIC, BIC, and aBIC, it also had the lowest entropy value, and the LMR *p*-value was not statistically significant (*P* > 0.05). Considering these results and the practical significance, the 3-category model was chosen as the optimal profile model for family management characteristics in children with RB.

**Table 1:** Comparison of parameter metrics in different potential profile models.

Model	AIC	BIC	aBIC	Entropy value	LMR (*P*)	BLRT (*P*)	Probability of a category
1	21,789.324	21,842.246	21,804.149	–	–	–	1
2	20,714.113	20,797.906	20,737.586	0.838	<0.001	<0.001	0.566, 0.434
3	20,359.227	20,473.891	20,391.347	0.844	<0.001	<0.001	0.444, 0.154, 0.402
4	20,241.525	20,387.061	20,282.294	0.819	0.057	<0.001	0.091, 0.328, 0.228, 0.353

AIC, Akaike information criterion; BIC, Bayesian information criterion; aBIC, adjusted Bayesian information criterion; LMR, Lo-Mendell-Rubin; BLRT, Bootstrap-based likelihood ratio test.

**Figure 1:**
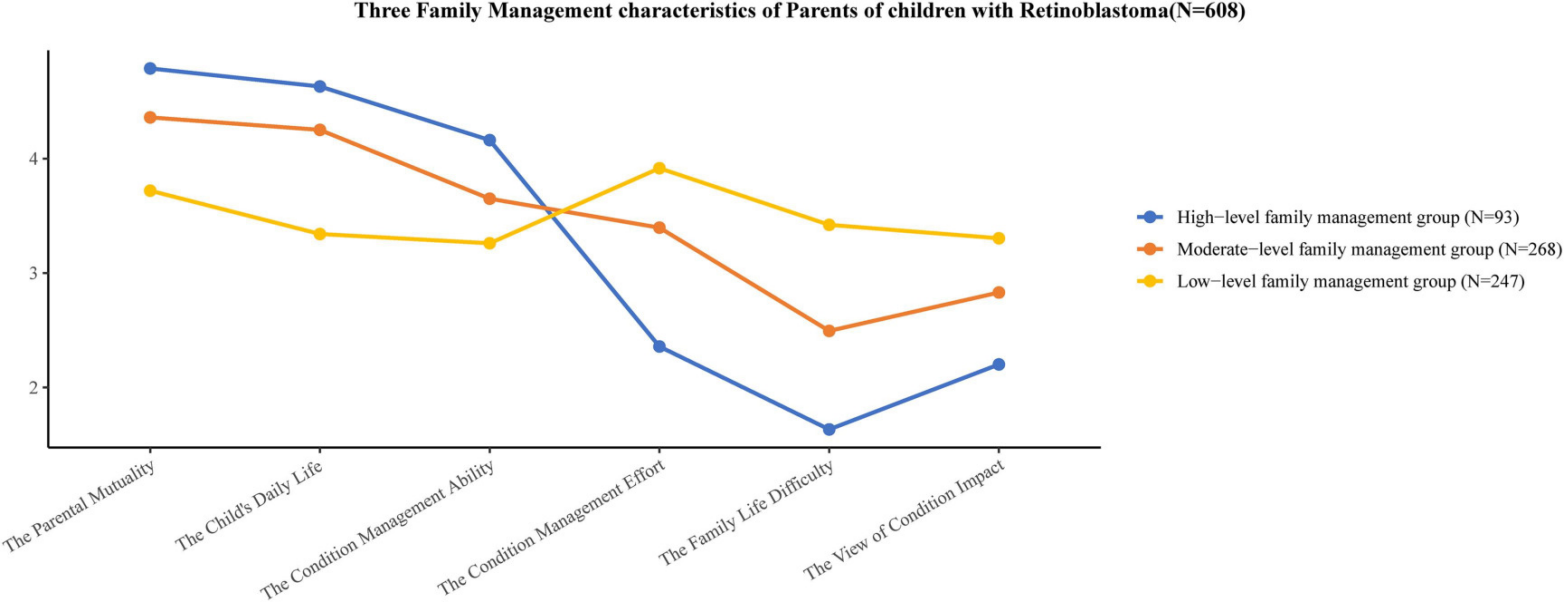
Three characteristics of family management Among parents of children with retinoblastoma. The vertical axis shows the standardised scores for the six FaMM sub-scales. The standardised scoring method is as follows: Standardised scores for each sub-scale = actual score for each sub-scale/number of items. The horizontal axis shows the names of FaMM's six subscales.

Based on the scores from the three categories of family management characteristics for children with RB across the six subscales of the FaMM, the categories were named (see Figure 1). Category 1 had higher scores than the other two groups in the three positive scales (Child's Daily Life, Condition Management Ability, and Parent Mutuality) and lower scores in the three negative scales (Condition Management Effort, Family Life Difficulty, and View of Condition Impact), leading to the designation of this group as the high-level family management group, comprising 93 (15.30%) cases. Category 2 scores fell between those of categories 1 and 3, with a relatively stable trend, so this group was named the moderate-level family management group, comprising 268 (44.07%) cases. Category 3 scored lower than the other two groups in the three positive dimensions and higher in the three negative dimensions, thus, it was designated as the low-level family management group, comprising 247 (40.63%) cases.

### 3.3 Univariate analysis of basic information and family management characteristics of children with RB and their parents

Univariate analysis results indicated statistically significant differences (*P* < 0.05) among the three categories of family management characteristics regarding the children's age, duration of disease, laterality of diseased eyes, family history of disease, duration of care, family functioning, comprehensive needs, residence, education level, type of family, caregiving type, coping tendencies, social support, depression, anxiety, and stress(see Table 2).

**Table 2:** Univariate analysis of family management characteristics among children with retinoblastoma and their parents (*N* = 608).

Variant	*N* (%)/*M* (IQR)	High-level family management group (*n* = 93)	Moderate-level family management group (*n* = 268)	Low-level family management group (*n* = 247)	Statistics	*P* values
Children						
Age (months)	48.10 (28.08, 73.15)	56.12 (35.11, 85.15)	48.10 (28.08, 75.09)	46.09 (27.06, 70.13)	6.772^#^	**0**.**034**
Duration of disease (months)	23.00 (9.00, 45.00)	30.00 (12.00, 53.00)	20.50 (9.75, 43.00)	22.00 (8.00, 41.00)	6.254^#^	**0**.**044**
Gender						
Male	319 (52.47)	53 (16.61)	129 (40.44)	137 (42.95)	3.670	0.160
Female	289 (47.53)	40 (13.84)	139 (48.10)	110 (38.06)		
Laterality of diseased eyes						
Unilateral	399 (65.62)	74 (18.55)	184 (46.12)	141 (35.33)	17.094	**<0**.**001**
Bilateral	209 (34.38)	19 (9.09)	84 (40.19)	106 (50.72)		
Family history of disease						
Yes	15 (2.47)	1 (6.67)	2 (13.33)	12 (80.00)	9.916*	**0**.**007**
No	593 (97.53)	92 (15.51)	266 (44.86)	235 (39.63)		
Parents						
Age (years)	34.36 (30.78, 37.36)	34.44 (30.53, 36.53)	34.44 (30.86, 37.30)	34.19 (30.99, 37.57)	0.669^#^	0.716
Duration of care (hours)	8.00 (4.00, 12.00)	6.00 (4.00, 10.00)	9.50 (4.75, 12.00)	8.00 (4.00, 12.00)	11.249^#^	**0**.**004**
Family functioning	8.00 (5.00, 10.00)	10.00 (8.00, 10.00)	9.00 (6.00, 10.00)	5.00 (4.00, 9.00)	95.067^#^	**<0**.**001**
Comprehensive needs	69.00 (52.00, 87.00)	54.00 (41.00, 66.00)	69.00 (52.00, 83.00)	81.00 (60.00, 97.00)	69.563^#^	**<0**.**001**
Social support	60.00 (48.00, 72.00)	71.00 (59.00, 80.00)	62.00 (51.00, 72.00)	54.00 (46.00, 66.00)	58.442^#^	**<0**.**001**
Depression	4.00 (1.00, 7.00)	0.00 (0.00, 2.00)	3.00 (1.00, 6.00)	7.00 (4.00, 11.00)	176.059^#^	**<0**.**001**
Anxiety	4.00 (1.00, 7.00)	1.00 (0.00, 2.00)	3.00 (1.00, 6.00)	6.00 (3.00, 11.00)	136.569^#^	**<0**.**001**
Stress	6.00 (3.00, 9.00)	1.00 (0.00, 4.00)	5.00 (2.00, 7.00)	8.00 (6.00, 12.00)	159.778^#^	**<0**.**001**
Relationship with children						
Mother	419 (68.91)	66 (15.75)	192 (45.82)	161 (38.43)	2.720	0.257
Father	189 (31.09)	27 (14.29)	76 (40.21)	86 (45.50)		
Residence						
Town	312 (51.32)	62 (19.87)	143 (45.83)	107 (34.30)	15.541	**<0**.**001**
Rural or township	296 (48.68)	31 (10.47)	125 (42.23)	140 (47.30)		
Education level						
Junior high school and below	157 (25.82)	11 (7.00)	63 (40.13)	83 (52.87)	30.329	**<0**.**001**
High school or junior college	134 (22.04)	14 (10.45)	58 (43.28)	62 (46.27)		
College and above	317 (52.14)	68 (21.45)	147 (46.37)	102 (32.18)		
Number of children						
1	258 (42.43)	43 (16.67)	116 (44.96)	99 (38.37)	7.531	0.110
2	270 (44.41)	45 (16.67)	110 (40.74)	115 (42.59)		
≥3	80 (13.16)	5 (6.25)	42 (52.50)	33 (41.25)		
Type of family						
Nuclear family	305 (50.16)	41 (13.44)	137 (44.92)	127 (41.64)	22.897	**<0**.**001**
Stem family	203 (33.39)	45 (22.17)	94 (46.30)	64 (31.53)		
Other types	100 (16.45)	7 (7.00)	37 (37)	56 (56)		
Caregiving type						
Independent care	182 (29.93)	15 (8.24)	77 (42.31)	90 (49.45)	13.616	**0**.**001**
Assisted care	426 (70.07)	78 (18.31)	191 (44.84)	157 (36.85)		
Availability of a spouse or live in companion						
No	92 (15.13)	10 (10.87)	38 (41.30)	44 (47.83)	2.962	0.227
Yes	516 (84.87)	83 (16.09)	230 (44.57)	203 (39.34)		
Coping tendencies						
Active response	276 (45.39)	58 (21.01)	139 (50.36)	79 (28.62)	33.254	**<0**.**001**
Negative response	332 (54.61)	35 (10.54)	129 (38.86)	168 (50.60)		

M (IQR), Median (Quartiles); The duration of care, i.e., the amount of time that the father or mother spent caring for the child in the past six months, assuming that the child required 12 h of care per day; In the test statistic values.

The bold values indicate statistical significance with *P* < 0.05.

# Kruskal–Wallis Test.

* Fisher Exact Test, all others are Pearson's and Chi-Square tests.

### 3.4 Results of multiple analysis of family management categories of children with RB

A multiple logistic regression analysis, using variables with significant differences (*p* < 0.05) from the univariate analysis as independent variables and family management categories as dependent variables (with the high-level family management groupas the reference). In comparing the moderate-level to the high-level family management group, high comprehensive needs (OR = 1.020, *P* = 0.001) were associated with the moderate-level family management group. High social support (OR = 0.978, *P* = 0.045) was more likely in the high-level family management group (see Table 3). Comparing the low-level to the high-level family management group showed that better family functioning (OR = 0.821, *P* = 0.004), unilateral diseased eyes (OR = 0.286, *P* = 0.001) and high social support (OR = 0.972, *P* = 0.023) were linked to the high-level family management group. However, severe depression (OR = 1.320, *P* = 0.005), severe stress (OR = 1.210, *P* = 0.033), high comprehensive needs (OR = 1.025, *P* = 0.001), junior high school and below education (OR = 4.021, *P* = 0.005) were more likely in the low-level family management group (see Table 4).

**Table 3:** Comparative analysis of family management between moderate-level and high-level groups.

Variable	*β*-values	Std. error	Wald	*P* values	OR-values (95% CI)
Constant term	1.044	1.053	0.982	0.322	—
Social support	−0.022	0.011	4.008	**0**.**045**	0.978 (0.957–1.000)
Comprehensive needs	0.020	0.006	10.591	**0**.**001**	1.020 (1.008–1.033)

OR, odds ratio; CI, confidence interval.

The bold values indicate statistical significance with *P* < 0.05.

**Table 4:** Comparative analysis of family management between low-level and high-level groups.

Variable	*β-*values	Std. error	Wald	*P* values	OR*-*values (95% CI)
Constant term	2.176	1.145	3.61	0.057	—
Family functioning	−0.197	0.069	8.253	**0**.**004**	0.821 (0.718–0.939)
Depression	0.278	0.098	8.016	**0**.**005**	1.320 (1.089–1.601)
Stress	0.191	0.089	4.555	**0**.**033**	1.210 (1.016–1.442)
Social support	−0.029	0.013	5.155	**0**.**023**	0.972 (0.948–0.996)
Comprehensive needs	0.024	0.007	11.652	**0**.**001**	1.025 (1.01–1.039)
Diseased Eyes					
Bilateral					
Unilateral	−1.252	0.373	11.289	**0**.**001**	0.286 (0.138–0.593)
Level of education					
College and above					
High school or junior college	0.664	0.445	2.225	0.136	1.943 (0.812–4.651)
Junior high school and below	1.392	0.491	8.042	**0**.**005**	4.021 (1.537–10.520)

OR, odds ratio; CI, confidence interval.

The bold values indicate statistical significance with *P* < 0.05.

## 4 Discussion

### 4.1 Characteristics of family management of parents of children with RB

This study defined three categories of parental family management for children with RB through latent profile analysis, highlighting heterogeneity in management styles. The categories are named as follows: high-level family management group (15.30%), moderate-level family management group (44.07%), and low-level family management group (40.63%). The predominant categories among parents of children with RB were the moderate-level and the low-level family management groups, indicating a generally low level of family management ability. This finding slightly contrasts with previous research (26), possibly due to the rarity of RB as a childhood eye cancer and the resulting lack of relevant scientific knowledge, which hampers the development of caregiving skills. In addition, as RB primarily affects infants and children under five, it often necessitates long-term parental care. Caregivers must frequently take their children to hospital for check-ups, which has a significant impact on both the children and their families.

### 4.2 Analysis of factors influencing family management characteristics of parents of children with RB

#### 4.2.1 Parents of children with RB with better family functioning or unilateral diseased eyes had a higher probability of belonging to the high-level family management group

According to literature, family functioning plays a vital role in the relationship between contextual influences and family management (10). Intra-family communication is key to improving patient and family outcomes. Openly sharing concerns and discussing intimacy and the burdens of the disease, for example, are effective communication strategies that can help families access resources and adopt positive coping mechanisms (8). The attributes of a nursing presence can be incorporated into a family-centred care framework, yielding positive clinical, social and emotional outcomes for patients and their families (27). Studies show that 57% of families of children with chronic illnesses have adopted a family-focused or somewhat family-focused management model, which leads to better family and child functioning compared to somewhat condition-focused or condition-focused models (12). The child- and family-focused care model significantly improves children's quality of life by emphasizing holistic care and supporting both the child and their family, enhancing the family's coping ability and disease management. Family members are encouraged to strengthen emotional communication, provide mutual support, and work together to overcome the challenges of the disease and treatment.

A childhood cancer diagnosis is an extremely serious blow for families. Informal care is vital for patients with cancer, and non-professional caregivers often face significant time commitments and social costs (28). A 32-year study of 6,859 fathers and 7,098 mothers of children diagnosed with cancer in Sweden found that parents of children with cancer experienced more physical health problems than parents of children without cancer. This may be due to the need for frequent medical treatment for their children (29). This survey found that 79.57% (74/93) of children in families with high management levels had unilateral retinoblastoma, which is significantly higher than the 57.09% (141/247) found in families with low management levels. This difference may be due to several factors. Unilateral cases tend to present with milder symptoms and offer more flexible treatment options, resulting in a lighter economic burden. They also interfere less with daily family life. Furthermore, caregivers have fewer concerns about the visual prognosis, appearance and future quality of life of children with unilateral retinoblastoma. They experience less psychological stress and find it easier to focus on current treatment and care. By contrast, bilateral retinoblastoma often requires the synchronous management of binocular lesions, involving high treatment complexity, long treatment cycles, and intensive follow-up (30). This significantly increases the difficulty and burden on families.

#### 4.2.2 Parents of children with RB with high social support had a higher probability of belonging to the high-level family management group

Social support is vital for families coping with illness, with support from peers, healthcare professionals, and social networks forming its core. In particular, ongoing support from peers and healthcare professionals is essential for building interpersonal relationships, creating support networks, and reducing psychological stress among family members (31). Research indicates that support from strangers and/or members of support groups who have experience with RB can be particularly valuable, sometimes even more so than support from healthcare professionals (32). Establishing and improving social support networks can provide more help and resources to families with children suffering from RB. Additionally, public awareness and education about RB should be strengthened. Pediatric oncology nurses and other healthcare professionals implementing family management programs can improve family management and enhance the quality of life for these children (33). Therefore, healthcare professionals should foster communication with caregivers, explain disease-related information in simple terms, and provide patients with clear instructions on care skills. Ongoing assessment of caregivers' understanding and timely feedback can significantly enhance the family's ability to cope with the disease and improve their resilience.

#### 4.2.3 Parents of children with RB with severe depression, severe stress and low literacy had a higher probability of belonging to the low-level family management group

Studies have shown that mothers of children with tumors experience significant psychological stress and mood swings, with a notable risk of deterioration in their quality of life (34). Higher anxiety levels among oncology caregivers are linked to an increased caregiving burden (35). A meta-analysis reported that the prevalence of anxiety, depression, and PTSD in parents of children with tumors is 21%, 28%, and 26%, respectively, consistently higher than in parents of children without tumors (36). In parents of children with RB, the reported prevalence of anxiety and depression was 41.32% and 29.97%, respectively (37). In this study, the prevalence of anxiety, depression, and stress among parents of children with RB was 50.50%, 45.70%, and 33.20%, respectively, slightly differing from previous reports, most likely due to variations in measurement tools and thresholds. Parental satisfaction with their child's tumor management mediates the relationship between family life difficulties, mutual support, and depression (38).

Lack of knowledge and financial constraints are key barriers to seeking timely treatment for RB symptoms (39). Parental literacy directly impacts family management (10). Some parents of children with RB experience difficulties in expressing their needs or accessing the information they desire due to psychological distress (32). Low literacy makes it difficult for these parents to communicate with medical professionals and understand medical terminology and treatment recommendations, which hinders their ability to manage the child's condition and adhere to treatment plans. Limited literacy also restricts their access to reliable medical information through the Internet or books, resulting in poor decision-making. Families with low literacy levels may also lack social resources and connections, limiting their ability to seek support and assistance from the community or charitable organizations, which further complicates family management.

## 5 Limitations

This study is a cross-sectional survey, meaning the data were collected at a single point in time. Therefore, it is not possible to infer the temporal order or causality between the exposure factors and outcomes directly, nor can the survey reflect dynamic changes in family management over time. Additionally, conducting the survey online may have excluded low-income groups lacking internet access, affecting the representativeness of the sample. Furthermore, the lack of direct supervision in the online questionnaire process may lead to arbitrary responses and a decline in data quality. The respondents in this study are the parents of affected children, whose views may not accurately reflect those of other family members. Other family members, such as affected children, grandparents and siblings, may have different viewpoints which should be considered to provide a more comprehensive picture of the current state of family management. The clinical features of RB, including stage (intraocular, orbital or metastatic), prescribed treatment modalities (enucleation alone, enucleation + chemotherapy, enucleation + chemotherapy + radiation) and treatment goals (palliative or curative), significantly increase the complexity of patient management and profoundly affect family care strategies and management methods. As these factors were not systematically included in this study, future research examining them as key variables is recommended to reveal the association between disease characteristics and family management more comprehensively. The survey was limited to three hospitals in the Shanghai area, which may limit the generalisability of the results. Future research could involve an international joint study to expand the sample size and validate the findings of this study further.

## 6 Conclusion

The family management characteristics of parents of children with RB exhibit group heterogeneity. This study categorised participants into three groups based on the results of the latent profile analysis: high-, moderate- and low-level family management. The high-level group accounted for only 15.30% of participants and consisted mainly of individuals who had initially been categorised as moderate or low, with a close ratio of 1:1 between these two groups. The study highlights significant disparities in management styles and identifies key influencing factors, including family functioning, comprehensive needs, educational background, depression, stress and social support. These results will be invaluable for developing precise intervention strategies in future. For example, specific measures could be implemented to address the factors that influence group heterogeneity, thereby improving family management and quality of life for children with RB and their families.

## Data Availability

The original contributions presented in the study are included in the article/Supplementary Material, further inquiries can be directed to the corresponding authors.
